# Impact of thromboprophylaxis timing and duration on thromboembolism and bleeding in metabolic bariatric surgery

**DOI:** 10.1093/bjsopen/zrag097

**Published:** 2026-07-21

**Authors:** Martin Löfling Skogar, Martina Azzurra Branciforte, Erik Stenberg

**Affiliations:** Department of Surgical Sciences, Uppsala University, Uppsala, Sweden; Department of Surgical Sciences, Uppsala University, Uppsala, Sweden; Department of Surgery, Faculty of Medicine and Health, Örebro University, Örebro, Sweden

**Keywords:** anticoagulants, low-molecular-weight heparin, heparin administration, venous thromboembolism, epidemiology

## Abstract

**Background:**

Patients undergoing metabolic bariatric surgery are at risk of both venous thromboembolism (VTE) and bleeding complications, but the optimal thromboprophylaxis strategy remains uncertain. Therefore, this retrospective cohort study evaluated perioperative outcomes depending on the perioperative thromboprophylaxis strategy

**Methods:**

This study used data from the validated nationwide Scandinavian Obesity Surgery Registry, including all primary bariatric procedures in Sweden between 2008 and 2023. Exposures were timing of initiation (preoperative, postoperative, other) and the duration of thromboprophylaxis (< 7, 7–10, 11–14, or > 14 days). The primary outcome was VTE; secondary outcomes were intraoperative and postoperative bleeding. Outcomes were analysed using logistic regression models, adjusting for demographic, clinical, and surgical covariates.

**Results:**

Among 83 801 patients (76.9% female, mean age 40.9 years, mean body mass index 41.8 kg/m^2^), the incidences of VTE, intraoperative bleeding, and postoperative bleeding were 0.1% (76 patients), 0.7% (577), and 1.6% (1373), respectively. No statistically significant associations were observed between the timing of thromboprophylaxis and VTE, or between thromboprophylaxis duration and either VTE or postoperative bleeding. Initiation of prophylaxis after surgery was associated with a lower risk of intraoperative bleeding than preoperative initiation (odds ratio 0.52; 95% confidence interval 0.37 to 0.74). Risk factors for VTE included previous VTE, intraoperative bleeding, and longer operating time, whereas risk factors for postoperative bleeding included higher age, male sex, cardiovascular co-morbidity, and antidepressant use.

**Conclusion:**

The overall incidence of thrombotic complications after metabolic bariatric surgery was low, whereas bleeding events were more common. Compared with preoperative thromboprophylaxis initiation, postoperative initiation was associated with reduced intraoperative bleeding, whereas no increase in VTE was detected.

## Teaser

This nationwide registry-based cohort study including over 83 000 bariatric procedures evaluated the impacts of the timing and duration of thromboprophylaxis on postoperative venous thromboembolism and bleeding risk. Neither timing nor duration significantly affected venous thromboembolism or postoperative bleeding rates, but postoperative thromboprophylaxis initiation was associated with a markedly lower risk of intraoperative bleeding compared with preoperative initiation. These findings suggest that starting thromboprophylaxis after surgery may provide a more favourable balance between thrombotic protection and bleeding risk in metabolic bariatric surgery.

## Introduction

Obesity is widely recognized as a significant risk factor for the development of symptomatic venous thromboembolic complications following surgery^1,2^. Multiple factors, such as a hypercoagulable state and reduced blood flow in the infradiaphragmatic region, are thought to contribute to the heightened risk of thrombus formation in patients with obesity^3,4^.

Metabolic bariatric surgery (MBS) has become a proven therapeutic option for the treatment of severe obesity^5^. Reflecting the increasing burden of obesity, the number of bariatric procedures has increased over time and is projected to continue increasing in coming years^6^. Currently, laparoscopic techniques are the standard approach in MBS. However, by increasing intra-abdominal pressure, laparoscopy can further decrease venous blood flow and increase the risk of thrombus formation^7^. The rate of venous thrombotic events (VTE) after MBS is low, but not negligible, ranging between 0.3% and 3%^8^. VTE has also been reported to be one of the main causes of early mortality after MBS^9^. However, bleeding complications are one of the most common serious perioperative complications in this group of patients, making it challenging to identify the optimal balance between thrombotic and bleeding risks^10^.

Current guidelines for preoperative and postoperative antithrombotic management of patients undergoing MBS advocate for anticoagulation as a strategy to limit the risk of postoperative thrombotic complications^8,11,12^. However, although the evidence in favour of using thromboprophylaxis is considered to be moderate to strong^11,12^, current recommendations on the timing and duration of treatment are mainly based on limited evidence or expert consensus^12^. Consequently, there is substantial variation in current clinical practice regarding the timing (that is, before or after surgery), dosage, and duration of anticoagulation administration in patients undergoing MBS. Therefore, the aim of this study was to assess risks of VTE and bleeding complications for different thromboprophylaxis strategies in patients undergoing MBS, using data from a large, nationwide registry.

## Methods

The study was preregistered with an analysis plan in an independent, institutional registry and is available at https://www.researchweb.org/oct/project/285054.

This was a retrospective registry-based study using prospectively collected data from the Scandinavian Obesity Surgery Registry (SOReg). SOReg was established in 2007 and, according to a validation study conducted in 2021^13^, included 97% of all bariatric procedures performed in Sweden, with data validity of 99%. As of December 2025, the registry contains data for more than 100 000 procedures.

For this study, data from 2008 to 2023 were used and included only primary bariatric procedures. The following data were extracted from the registry: patient characteristics (age, sex, height, weight, co-morbidities), surgical data (date and type of procedure, operative time, thromboprophylaxis administered), and follow-up data 6 weeks after surgery (occurrence of thromboembolic events and/or bleeding complications). The 6-week follow-up period corresponds to the standardized postoperative follow-up interval in SOReg and captures the majority of clinically relevant postoperative thromboembolic and bleeding events^14,15^.

To ensure valid assessment of cardiovascular co-morbidity, data from SOReg were cross-linked with the National Inpatient Register. Cardiovascular diagnoses were identified using International Classification of Diseases, Tenth Revision (ICD-10) codes I20–I22 (ischaemic heart disease), I47–I48 (atrial fibrillation and other arrhythmias), and I50 (heart failure).

### Exposure

The primary exposure was defined along two dimensions: the timing and duration of thromboprophylaxis. The timing of initiation was categorized as either preoperative (that is, the day before surgery or on the day of surgery before the start of surgery), postoperative (that is, on the day of surgery after the start of surgery), or other start time (predefined registry category; timing not further specified). The duration of thromboprophylaxis was categorized as either a short course (< 7 days), a medium–long course (7–10 days), a long course (11–14 days), or a very long course (> 14 days).

The variables were gradually introduced in the registry and thus data were not available for all patients in the registry.

### Outcome

The primary outcome was VTE, defined as clinically diagnosed, symptomatic deep vein thrombosis (DVT) and/or pulmonary embolism (PE) recorded in the registry within 6 weeks after surgery (events reflect diagnoses made in routine clinical practice and are not based solely on patient self-report). Secondary outcomes were intraoperative bleeding, defined as blood loss > 100 ml and/or documented by the operating surgeon as an intraoperative complication requiring active intervention, and postoperative bleeding complications, defined as Clavien–Dindo (CD) grade I or higher^16^, occurring within the same 6-week postoperative period. Analyses of intraoperative bleeding primarily focused on timing of thromboprophylaxis initiation, whereas analyses of postoperative bleeding focused on the duration of thromboprophylaxis. This approach was based on the hypothesis that the timing of initiation has a greater effect on intraoperative bleeding risk, whereas duration represents a more biologically plausible determinant of postoperative bleeding.

### Covariates

Covariates were selected based on clinical relevance and previous reported associations with VTE or bleeding risk and were: age, sex, body mass index (BMI), cardiovascular co-morbidities (medication use and/or ICD-10 codes for ischaemic heart disease, atrial fibrillation/other arrhythmias, or heart failure), depression, hypertension, dyslipidaemia, and diabetes (defined in SOReg as present if the patient was receiving active pharmacological treatment for that specific disease); a history of DVT/PE; smoking history; and surgical access (laparoscopic/open), bariatric procedure type, intraoperative bleeding (> 100 ml blood loss and/or documented by the operating surgeon as a bleeding complication), operative time, and surgical year. To explore potential institutional variation, descriptive analyses stratified by centre procedural volume were performed. Centres were categorized according to the total number of procedures performed (< 1000, 1000–2000, > 2000) during the study period.

### Statistical analysis

Independent-sample *t* tests and χ^2^ tests were used as univariate analyses for continuous and categorical variables, respectively. To assess the association between the thromboprophylaxis regimen and the risk of VTE and bleeding complications, univariable and multivariable logistic regression models were constructed, with results presented as odds ratio (ORs) with their 95% confidence interval (95% c.i.). All analyses were conducted using complete case analysis for the variables included in each model. Patients with missing data for a given exposure, outcome, or covariate were excluded from that specific analysis; no imputation was performed. Covariates were selected based on a combination of statistical significance in univariable analyses (*P* < 0.05) and clinical relevance supported by previous literature. This approach ensured adjustment for both empirically and theoretically important confounders while taking into account the limited number of VTE events. To account for potential clustering effects arising from the multicentre design, multilevel logistic regression with centre identity as a random intercept was attempted for both bleeding outcomes; however, models consistently failed to achieve stable convergence across multiple specifications, as indicated by a non-positive definite Hessian matrix. As an alternative, centre procedural volume was included as a fixed covariate, categorized as < 1000, 1000–2000, and > 2000 procedures, which was found to be significantly associated with both bleeding outcomes in univariable analysis (*P* < 0.001; *Table S1*) and was therefore retained in the multivariable models.

All *P* values are 2-sided and *P* < 0.05 was considered statistically significant. With the observed sample size, the study had 80% power (α = 0.05) to detect absolute differences in VTE incidence of approximately 0.2% between groups. All statistical analyses were conducted using SPSS^®^ version 30 (IBM, Armonk, NY, USA).

### Ethical considerations

This study was approved the Swedish Ethical Review Authority (Dnr 2024-01662-01).

## Results

In all, 83 801 individuals were identified in the SOReg, of whom 76.9% were female, with a mean(standard deviation) age of 40.9(11.2) years and a mean BMI of 41.8(5.8) kg/m^2^. Patients had undergone either primary gastric bypass (77.3%), sleeve gastrectomy (22.1%), or duodenal switch (0.6%), of which 97.7% were completed as laparoscopic procedures. Data for the two exposure variables (that is, the timing of initiation and the duration of thromboprophylaxis) were available for 33 058 patients (39.4%) and 50 722 patients (60.5%), respectively (*Table 1*). Follow-up data at 6 weeks for the two outcomes (VTE and postoperative bleeding) were available for 96.0% of patients (80 474 and 80,477 patients, respectively), whereas data on intraoperative bleeding were available for 100% of patients. History of DVT/PE and smoking status had moderate levels of missing data (9.7% and 7.9%, respectively), whereas all other covariates had near-complete capture (*Table 1*). Compared with the total cohort, both subcohorts (that is, thromboprophylaxis timing and duration) had a higher proportion of females, lower BMI, a higher prevalence of obstructive sleep apnoea syndrome, more smokers, more laparoscopic procedures, a higher share of more recent surgery years, and differences in bariatric procedure distribution (all *P* < 0.05). In addition, compared with the total cohort, the timing subcohort had a higher prevalence of depression and was more concentrated at higher-volume centres, whereas the duration sub-cohort had a higher prevalence of hypertension and a greater representation of lower-volume centres (all *P* < 0.05).

**Table 1:** Baseline characteristics of the entire cohort and of the two subcohorts with available data on the timing of initiation and duration of thromboprophylaxis

	Total cohort (*n* = 83 801)	No. missing data (%)	Timing cohort (*n* = 33 058)	Duration cohort (*n* = 50 722)
Age (years), mean(s.d.)	40.9(11.2)	0 (0.0%)	40.8(11.3)	41.0(11.2)
Female	64 447 (76.9%)	0 (0.0%)	25 923 (78.4%)	39 150 (77.2%)
BMI (kg/m^2^), mean(s.d.)	41.8(5.8)	117 (0.1%)	41.1(5.8)	41.5(5.6)
Cardiovascular disease	3681 (4.4%)	0 (0.0%)	1364 (4.1%)	2163 (4.3%)
OSAS	8325 (9.9%)	124 (0.1%)	3392 (10.3%)	5456 (10.8%)
Hypertension	20 515 (24.5%)	124 (0.1%)	7982 (24.1%)	12 918 (25.5%)
Diabetes	11 063 (13.2%)	124 (0.1%)	4016 (12.1%)	6602 (13.0%)
Dyslipidaemia	7788 (9.3%)	124 (0.1%)	2979 (9.0%)	4749 (9.4%)
Depression	13 555 (16.2%)	124 (0.1%)	6037 (18.3%)	8234 (16.2%)
History of DVT/PE	1823 (2.2%)	8 132 (9.7%)	773 (2.3%)	1159 (2.3%)
History of smoking	21 977 (26.2%)	6 606 (7.9%)	9216 (27.9%)	15 277 (30.1%)
Laparoscopic surgery	81 878 (97.7%)	2 (0.0%)	32 929 (99.6%)	50 015 (98.6%)
**Bariatric procedure**		0 (0.0%)		
Gastric bypass	64 760 (77.3%)	0 (0.0%)	19 315 (58.4%)	36 746 (72.4%)
Sleeve gastrectomy	18 515 (22.1%)	0 (0.0%)	13 645 (41.3%)	13 665 (26.9%)
Duodenal switch	526 (0.6%)	0 (0.0%)	98 (0.3%)	311 (0.6%)
**Surgical year period**		0 (0.0%)		
2008–2013	37 648 (44.9%)	0 (0.0%)	4 (0.0%)	17 121 (33.8%)
2014–2018	28 421 (33.9%)	0 (0.0%)	17 740 (53.7%)	19 822 (39.1%)
2019–2023	17 732 (21.2%)	0 (0.0%)	15 314 (46.3%)	13 779 (27.2%)
**Centre procedural volume**		0 (0.0%)		
< 1000 procedures	13 070 (15.6%)	0 (0.0%)	4619 (14.0%)	10 089 (19.9%)
1000–2000 procedures	20 762 (24.8%)	0 (0.0%)	7477 (22.6%)	14 674 (28.9%)
> 2000 procedures	49 969 (59.6%)	0 (0.0%)	20 962 (63.4%)	25 959 (51.2%)

Values are *n* (%) unless otherwise stated. The variables were gradually introduced into the registry and so are not available for all patients in the registry. Missing data are reported per variable in the total cohort. Subcohorts differ from the total cohort, with higher proportion of females, more laparoscopic procedures, more recent surgery years, lower BMI, a higher prevalence of OSAS, and more smokers, as well as differences in the distribution of bariatric procedures and centre procedural volume (all *P* < 0.05). The timing subcohort had a higher prevalence of depression, whereas the duration subcohort had a higher prevalence of hypertension (all *P* < 0.05). s.d., standard deviation; BMI, body mass index; OSAS, obstructive sleep apnoea syndrome; DVT, deep vein thrombosis; PE, pulmonary embolism.

Thromboprophylaxis consisted of low-molecular-weight heparins (for example, enoxaparin or dalteparin) in 92.3% of patients (46 805), although regimens varied widely. Thromboprophylaxis initiation occurred before surgery in 23.5% of patients (7760), after surgery in 57.9% of patients (19 130), and at other times in 18.7% of patients (6168). Thromboprophylaxis duration was a short course (< 7 days) in 9.3% of patients (4692), a medium–long course (7–10 days) in 69.1% of patients (35 052), a long course (11–14 days) in 18.9% of patients (9567), and a very long course (> 14 days) in 2.8% of patients (1411). Thromboprophylaxis initiation and duration also varied significantly across centres with different procedural volumes (*Table S2*).

### Venous thromboembolism

The overall incidence of VTE was 0.1% (76 patients) in the whole cohort, 0.1% (18) in the timing subcohort, and 0.1% (42) in the duration subcohort. No significant differences in VTE rates were observed according to either the timing of initiation (preoperative 0.1% (5 patients), postoperative 0.0% (7), and other 0.1% (6); *P* = 0.121), or the duration of thromboprophylaxis (short course 0.1% (4), medium–long course 0.1% (29), long course 0.1% (6), and very long-course 0.2% (3); *P* = 0.334).

Given the low number of events in the timing subcohort, multivariable regression analysis was not considered statistically justified. Multivariable analysis of thromboprophylaxis duration showed no association with the risk of VTE. A history of DVT/PE, the occurrence of intraoperative bleeding, and longer operating time were associated with an increased risk of VTE (*Table 2*).

**Table 2:** Univariable and multivariable regression analyses assessing associations between different variables and the risk of venous thromboembolism

	Univariable		Multivariable	
	OR	*P*	OR	*P*
**Duration of thromboprophylaxis**				
< 7 days	0.99 (0.35, 2.82)	0.985	0.84 (0.29, 2.39)	0.740
7–10 days	Reference		Reference	
11–14 days	0.73 (0.30, 1.76)	0.485	0.61 (0.25, 1.49)	0.278
> 14 days	2.53 (0.77, 8.32)	0.126	1.49 (0.43, 5.19)	0.527
History of DVT/PE	4.82 (2.20, 10.60)	< 0.001	5.19 (1.98, 13.60)	< 0.001
Intraoperative bleeding	9.93 (4.00, 24.70)	< 0.001	5.71 (1.31, 24.80)	0.020
Surgery time (per 1-min increase)	1.01 (1.01, 1.02)	< 0.001	1.008 (1.00, 1.02)	0.015
Age (per 1-year increase)	1.01 (0.99, 1.04)	0.322		
BMI (per 1-kg/m^2^ increase)	0.99 (0.91, 1.03)	0.976		
Male sex	1.18 (0.71, 1.97)	0.519		
Hypertension	1.41 (0.87, 2.28)	0.166		
Diabetes	1.73 (1.00, 3.00)	0.061		
Depression	0.98 (0.53, 1.82)	0.953		
History of smoking	0.77 (0.43, 1.36)	0.362		
Cardiovascular disease	2.19 (1.01, 4.77)	0.043		
OSAS	2.02 (1.13, 3.62)	0.015		
Dyslipidaemia	2.17 (1.22, 3.88)	0.007		
Surgical year	0.88 (0.83, 0.94)	< 0.001		
Laparoscopic surgery	0.12 (0.06, 0.21)	< 0.001		
**Bariatric procedure**				
Gastric bypass	Reference			
Sleeve gastrectomy	0.75 (0.41, 1.37)	0.354		
Duodenal switch	1.93 (0.27, 13.9)	0.516		

Values in parentheses are 95% confidence intervals. The number of covariates included in the multivariable model was limited due to the low number of events (42). OR, odds ratio; DVT, deep vein thrombosis; PE, pulmonary embolism; min, minutes; BMI, body mass index; OSAS, obstructive sleep apnoea syndrome.

### Intraoperative bleeding

The overall incidence of intraoperative bleeding was 0.7% (577 patients) in the whole cohort and 0.5% (157) in the timing subcohort. The incidence of intraoperative bleeding was significantly lower among patients who started thromboprophylaxis after surgery (0.4%, 75) or at other times (0.2%, 13) than among those who started thromboprophylaxis before surgery (0.9%, 69; *P* < 0.001).

Multivariable regression analysis showed that initiation of thromboprophylaxis after surgery was associated with approximately half the risk of intraoperative bleeding compared with initiation before surgery (OR 0.52; 95% c.i. 0.37 to 0.74; *P* < 0.001). In addition, higher BMI, higher age, the use of antidepressants, and sleeve gastrectomy (*versus* primary gastric bypass) were associated with increased risk, whereas laparoscopic surgery (*versus* open surgery) and surgery performed later in the study period were associated with decreased risk (*Table 3*).

**Table 3:** Univariable and multivariable regression analyses assessing associations between different variables and the risk of intraoperative bleeding

	Univariable		Multivariable	
	OR	*P*	OR	*P*
**Timing of initiation**				
Before	Reference		Reference	
After	0.44 (0.32, 0.61)	< 0.001	0.52 (0.37, 0.74)	< 0.001
Other	0.24 (0.13, 0.43)	< 0.001	0.37 (0.20, 0.70)	0.002
BMI (per 1-kg/m^2^ increase)	1.05 (1.04, 1.07)	< 0.001	1.03 (1.00, 1.06)	0.033
OSAS	1.43 (1.11, 1.85)	0.006	0.86 (0.53, 1.40)	0.546
Dyslipidaemia	1.78 (1.39, 2.28)	< 0.001	1.42 (0.88, 2.32)	0.154
Surgical year	0.86 (0.83, 0.88)	< 0.001	0.89 (0.83, 0.96)	0.001
Laparoscopic surgery	0.04 (0.03, 0.05)	< 0.001	0.08 (0.04, 0.16)	< 0.001
Age (per 1-year increase)	1.02 (1.01, 1.03)	< 0.001	1.03 (1.01, 1.05)	< 0.001
Male sex	1.36 (1.11, 1.65)	0.003	1.01 (0.68, 1.49)	0.972
Hypertension	1.74 (1.45, 2.10)	< 0.001	1.29 (0.87, 1.91)	0.213
Diabetes	1.66 (1.31, 2.10)	< 0.001	0.98 (0.62, 1.57)	0.946
Depression	1.67 (1.34, 2.08)	< 0.001	1.70 (1.18, 2.45)	0.004
History of smoking	1.27 (1.04, 1.55)	0.019	0.86 (0.60, 1.22)	0.391
**Bariatric procedure**				
Gastric bypass	Reference		Reference	
Sleeve gastrectomy	0.94 (0.75, 1.18)	0.578	1.84 (1.32, 2.57)	< 0.001
Duodenal switch	6.66 (4.05, 10.9)	< 0.001	0.48 (0.09, 2.38)	0.352
**No. of procedures**				
< 1000	Reference		Reference	
1000–2000	0.92 (0.73, 1.15)	0.463	1.34 (0.81, 2.20)	0.255
> 2000	0.53 (0.43, 0.66)	< 0.001	0.77 (0.48, 1.24)	0.281
Cardiovascular disease	1.25 (0.84, 1.85)	0.280		
History of DVT/PE	0.76 (0.39, 1.48)	0.425		
Surgery time (per 1-min increase)	1.021 (1.02, 1.02)	< 0.001		

Values in parentheses are 95% confidence intervals. Surgery time was excluded from multivariable analyses due to potential confounding by indication. OR, odds ratio; BMI, body mass index; OSAS, obstructive sleep apnoea syndrome; DVT, deep vein thrombosis; PE, pulmonary embolism; min, minutes.

### Postoperative bleeding

The overall incidence of postoperative bleeding was 1.6% (1373) in the entire cohort. Of these events, 9.0% (125) were classified as CD grade I, 30.4% (417) were classified as CD grade II, 4.2% (57) were classified as CD grade IIIa, and 44.0% (604) were classified as CD grade IIIb. Severe complications requiring intensive care (CD grades IVa–b) were uncommon (2.9%, 145), and mortality (CD grade V) was rare (0.4%, 25). In the duration subcohort, the incidence of postoperative bleeding was 1.7% (801), with no significant differences observed across thromboprophylaxis duration categories (short course 1.5% (70); medium–long course 1.6% (536); long course 1.7% (163); and very long course 2.4% (32); *P* = 0.334). Multivariable regression analysis yielded similar results, showing no association between thromboprophylaxis duration and the risk of postoperative bleeding. Older age, male sex, the use of antihypertensive medications or antidepressants, and cardiovascular disease were associated with an increased risk of postoperative bleeding, whereas surgery performed later in the time period was associated with a decreased risk (*Table 4*).

**Table 4:** Univariable and multivariable regression analyses assessing associations between different variables and the risk of postoperative bleeding

	Univariable		Multivariable	
	OR	*P*	OR	*P*
**Duration of thromboprophylaxis**				
< 7 days	0.96 (0.74, 1.24)	0.740	0.82 (0.63, 1.07)	0.146
7–10 days	Reference		Reference	
11–14 days	1.06 (0.89, 1.28)	0.507	0.94 (0.77, 1.14)	0.530
> 14 days	1.35 (0.92, 1.98)	0.129	1.33 (0.90, 1.97)	0.159
BMI (per 1-kg/m^2^ increase)	1.00 (0.99, 1.02)	0.559	0.99 (0.98, 1.01)	0.203
OSAS	1.48 (1.21, 1.80)	< 0.001	1.01 (0.82, 1.25)	0.919
Dyslipidaemia	1.60 (1.30, 1.96)	< 0.001	1.00 (0.79, 1.27)	0.987
Surgical year	0.94 (0.92, 0.96)	< 0.001	0.95 (0.93, 0.97)	< 0.001
Laparoscopic surgery (yes/no)	0.46 (0.30, 0.70)	< 0.001	0.60 (0.35, 1.02)	0.057
Age (per 1-year increase)	1.02 (1.01, 1.03)	< 0.001	1.01 (1.00, 1.02)	0.034
Male sex	1.87 (1.61, 2.18)	< 0.001	1.67 (1.42, 1.97)	< 0.001
Hypertension	1.74 (1.50, 2.02)	< 0.001	1.31 (1.09, 1.57)	0.005
Diabetes	1.52 (1.26, 1.83)	< 0.001	1.02 (0.83, 1.27)	0.827
Depression	1.18 (0.98, 1.42)	0.076	1.29 (1.07, 1.55)	0.009
History of smoking	1.17 (1.01, 1.36)	0.041	1.12 (0.96, 1.31)	0.144
**Bariatric procedure**				
Gastric bypass	Reference		Reference	
Sleeve gastrectomy	0.72 (0.60, 0.86)	< 0.001	0.98 (0.80, 1.20)	0.828
Duodenal switch	1.33 (0.62, 2.82)	0.464	0.85 (0.35, 2.06)	0.721
Cardiovascular disease	2.05 (0.58, 2.67)	< 0.001	1.38 (1.04, 1.82)	0.026
Surgery time (per 1-min increase)	1.01 (1.00, 1.01)	< 0.001	1.00 (1.00, 1.00)	0.233
**No. of procedures**				
< 1000	Reference		Reference	
1000–2000	0.98 (0.84, 1.15)	0.838	1.09 (0.88, 1.35)	0.416
> 2000	0.74 (0.64, 0.86)	< 0.001	0.91 (0.75, 1.12)	0.372
History of DVT/PE	1.43 (0.95, 2.14)	0.084		

Values in parentheses are 95% confidence intervals. OR, odds ratio; BMI, body mass index; OSAS, obstructive sleep apnoea syndrome; min, minutes; DVT, deep vein thrombosis; PE, pulmonary embolism.

## Discussion

In this large nationwide registry-based cohort study including more than 83 000 bariatric procedures, the overall incidence of VTE (0.1%) and bleeding complications (intraoperative 0.7%; postoperative 1.6%) was low. No statistically significant associations were detected between the timing or duration of thromboprophylaxis and VTE; however, the low absolute number of events limits the ability to exclude modest differences between thromboprophylaxis strategies (susceptibility to type II error). Nevertheless, postoperative initiation of thromboprophylaxis was associated with significantly reduced intraoperative bleeding, with no increase in VTE detected. Future studies should ideally include prospective designs with standardized thromboprophylaxis regimens and formal risk stratification, and should evaluate outcomes across different healthcare settings and populations. However, given the very low incidence of VTE observed after MBS, traditional randomized clinical trials may be difficult to conduct. In the absence of such trials, the findings from this large nationwide cohort study support postoperative initiation of thromboprophylaxis as a safe and effective strategy.

Despite a relatively high prevalence of cardiovascular risk factors in the present cohort (arterial hypertension > 20%, diabetes and dyslipidaemia ∼10%), the rate of thrombotic complications after MBS was low. This finding is consistent with a large systematic review reporting a 30-day incidence of thrombotic complications after MBS of only 0.5% after MBS^17^. In the present study, the only preprocedural covariate associated with an increased risk of VTE was previous VTE, which was associated with to a fivefold increased risk. Other studies^18,19^ have identified previous VTE as one of the strongest predictors of recurrence, particularly when exposed to high-risk settings such as surgery, thereby identifying a clinical subset of MBS patients in whom more aggressive or prolonged antithrombotic therapy may be beneficial. However, despite the large cohort, the number of VTE events in the present study was too small to allow for further analysis of this subgroup of patients.

In contrast, bleeding complications were considerably more common in the present study, occurring at rates approximately 10- to 15-fold higher than those of VTE. However, the postoperative bleeding rate in this study (1.6%) was nearly identical to that of a large Dutch cohort^20^ of almost 60 000 patients (1.5%), which, like the present study, also identified male sex, age, and cardiovascular disease as risk factors for bleeding complications.

Importantly, preprocedural anticoagulation was associated with an increased risk of intraoperative bleeding, whereas the risk of postoperative bleeding was not affected by the duration of anticoagulation. Therefore, given the overall low incidence of thrombotic complications observed in the present cohort and in previous studies, routine preprocedural anticoagulation for the prevention of VTE in patients undergoing MBS may disproportionately increase bleeding risk without providing meaningful VTE protection. Preoperative thromboprophylaxis in Sweden has historically been used selectively in patients perceived to be at increased thrombotic risk, with initiation typically occurring on the day before or the morning of surgery. In clinical practice, patients undergoing bariatric surgery have often been considered at higher baseline thrombotic risk due to severe obesity and associated risk factors, which has contributed to the use of preoperative prophylaxis in some centres and in some time periods. However, this approach is not uniform and has largely reflected local practice patterns rather than a universally standardized or internationally established regimen. However, due to a complex pathophysiology resulting in a prothrombotic state caused by obesity, in combination with the effects of intraoperative increased abdominal pressure and the anti-Trendelenburg position during surgery, patients undergoing MBS remain at high risk of VTE, and so providing thromboprophylaxis within 24 hours after surgery in combination with intermittent pneumatic leg compression remains recommended to reduce the risk of VTE during recovery^11,12,21,22^.

The results of the present study also highlight the complex interplay between bleeding and thrombosis in MBS patients. Intraoperative bleeding was associated with an increased risk of VTE in the study cohort. Several mechanisms may explain this association, including the need for transfusion of blood products, which is known to increase thrombosis risk, and the temporary discontinuation of antithrombotic therapy in the early postoperative phase (that is, withdrawal of protection)^23,24^. Overall, the interdependent nature of bleeding and thrombosis risks underscores the importance of prioritizing bleeding-avoidance strategies as the primary therapeutic goal in most patients undergoing MBS.

Finally, an interesting finding of this analysis is that both a laparoscopic approach and a more recent year of the index surgery were associated with a lower risk of bleeding. This likely reflects the broader adoption and increasing technical standardization of laparoscopic MBS in Sweden, in combination with improved preoperative preparation and enhanced perioperative care^25,26^.

This study is limited by its retrospective registry design, which carries risks of residual confounding, missing data, and misclassification. To mitigate these issues, analyses were adjusted for known confounders and validated high-quality sources of data were used from nationwide registries, including the SOReg, providing validated nationwide data with high coverage. Despite the high quality of the data, causal inference remains limited. Complete case analysis was used, which assumes data are missing completely at random; if this assumption is not fully met, some degree of bias cannot be excluded. Because data on thromboprophylaxis timing and duration were not available for all patients and were introduced progressively in the registry, analyses were restricted to subcohorts with complete exposure data. This may introduce selection bias and limits generalizability, particularly to earlier periods of bariatric practice. Another limitation is that the analyses did not formally account for clustering of patients within hospitals. Although multilevel modelling was attempted to account for clustering, stable convergence could not be achieved, as described in the Statistical Analysis section. In addition, detailed data on low-molecular-weight heparin dosing, weight-based or renal adjustments, and the use of mechanical prophylaxis were not available. Although cardiovascular co-morbidities were ascertained through cross-linkage with the National Inpatient Register using ICD-10 codes for ischaemic heart disease, atrial fibrillation, and heart failure, data on peripheral arterial disease (ICD-10 codes I70–I79) were not available and therefore could not be included as a covariate, representing a potential source of residual confounding. In addition, patients on chronic anticoagulation therapy (who may have different VTE and bleeding risks and for whom standard thromboprophylaxis may not apply) could not be identified, representing a potential source of residual confounding. Variability in these factors may have contributed to heterogeneity within exposure groups and reduced the ability to detect differences between strategies. Furthermore, the granularity of thromboprophylaxis timing data is limited by the registry structure. Preoperative timing is captured only as administration the day before or the day of surgery before incision, without exact hours, precluding analyses of clinically relevant intervals and limiting conclusions regarding the effect of specific timing on intraoperative bleeding risk. Similarly, postoperative timing is recorded only as administration on the day of surgery after the start of the procedure, without information on the exact timing (hours) after surgery. Although the validity of symptomatic VTE and bleeding are very high, the true incidence of VTE may be underestimated because it was based on follow-up data recorded at a 6-week follow-up rather than systematic clinical or radiological assessment of all patients. The very low incidence of VTE observed in this cohort reflects contemporary bariatric practice but poses inherent challenges for risk modelling. The limited event counts constrain multivariable adjustment and increase the likelihood of type II error. To mitigate overfitting, covariates with strong clinical relevance and statistical significance were prioritized in univariable analyses. Although this approach may not account for all possible confounders, it provides a balanced assessment of the association between thromboprophylaxis duration and VTE risk within the constraints of the data set used in this study.

In this large nationwide cohort study of more than 83 000 MBS patients, the overall incidence of VTE was very low (0.1%), whereas bleeding complications were more frequent (intraoperative 0.7%; postoperative 1.6%). No statistically significant associations were observed between the timing of thromboprophylaxis and VTE, or between the duration of thromboprophylaxis and either VTE or postoperative bleeding; however, initiation of prophylaxis after surgery was associated with an approximately halved risk of intraoperative bleeding compared with preoperative initiation. These findings suggest that postoperative initiation of thromboprophylaxis may offer a safer balance between thrombotic protection and bleeding risk in MBS.

## Supplementary Material

zrag097_Supplementary_Data

## Data Availability

Access to the data used in this study may be granted upon reasonable request to the corresponding author after approval from the SOReg steering committee and relevant ethical review boards.
