# Supplementary material for: Impact of thromboprophylaxis timing and duration on thromboembolism and bleeding in metabolic bariatric surgery
Source: BJS Open. 2026 Jul 21;10(4):zrag097. doi: 10.1093/bjsopen/zrag097 (PMC13387167; doi:10.1093/bjsopen/zrag097)
Supplement: zrag097_Supplementary_Data [file zrag097_supplementary_data.docx]

**Thromboprophylaxis Timing and Duration in Metabolic Bariatric Surgery: Impact on Thromboembolism and Bleeding**

Martin Löfling Skogar, MD PhD ^1^; Martina Azzurra Branciforte, MD ^1^; Erik Stenberg, MD, PhD ^2^

^1^ Department of Surgical Sciences, Uppsala University, Sweden

^2^ Department of Surgery, Faculty of Medicine and Health, Örebro University, Sweden

**Corresponding author.** Martin Löfling Skogar, martin.skogar@uu.se, **ORCID ID**: **https://orcid.org/0000-0002-6840-2749**

**Supplementary Materials - Index**

| **Supplementary Methods** |  |
| --- | --- |
| N/A | *pag. X* |
| N/A | *pag. Y* |
| **Supplementary Results** |  |
| N/A | *pag. X* |
| N/A | *pag. Y* |
| **Supplementary Appendixes** |  |
| N/A | *pag. X* |
| N/A | *pag. Y* |
| **Supplementary Figures and Tables** |  |
| Supplementary Table S1 and S2 | *pag. 5* |
| Detail | *pag. Y* |
| **References** | *pag. Z* |
|  |  |

**Supplementary Methods**

**Supplementary Results**

**Supplementary Appendixes**

**Supplementary Figures and Tables**

Supplementary Table S1. Association between center procedural volume and rates of venous thromboembolism (VTE) and bleeding complications after metabolic bariatric surgery. Centers were categorized based on total procedural volume during the study period.

| **Outcome** | **<1000 procedures** | **1000–2000 procedures** | **>2000 procedures** | **p-value** |
| --- | --- | --- | --- | --- |
| **Intraoperative bleeding** | 1.0% (128/13,070) | 0.9% (187/20,762) | 0.5% (262/49,969) | <0.001 |
| **Postoperative bleeding** | 2.0% (248/12,247) | 2.0% (401/20,129) | 1.5% (724/48,101) | <0.001 |
| **VTE (6 weeks)** | 0.14% (17/12,247) | 0.09% (19/20,129) | 0.08% (40/48,098) | 0.20 |

Supplementary Table S2. Variation in thromboprophylaxis timing and duration according to center procedural volume.

| **Timing of initiation** | **<1000** | **1000–2000** | **>2000** | **p-value** |
| --- | --- | --- | --- | --- |
| Preoperative | 11.5% | 41.1% | 47.4% | <0.001 |
| Postoperative | 13.7% | 21.7% | 64.6% |  |
| Other timing | 17.9% | 2.1% | 80.0% |  |
| **Duration** | **<1000** | **1000–2000** | **>2000** | **p-value** |
| <7 days | 8.2% | 60.3% | 31.5% | <0.001 |
| 7–10 days | 22.4% | 27.7% | 49.9% |  |
| 11–14 days | 14.4% | 19.4% | 66.3% |  |
| >14 days | 34.6% | 19.0% | 46.4% |  |

**References**
